# QTL Validation and Development of SNP-Based High Throughput Molecular Markers Targeting a Genomic Region Conferring Narrow Root Cone Angle in Aerobic Rice Production Systems

**DOI:** 10.3390/plants10102099

**Published:** 2021-10-03

**Authors:** Ricky Vinarao, Christopher Proud, Peter Snell, Shu Fukai, Jaquie Mitchell

**Affiliations:** 1 School of Agriculture and Food Sciences, The University of Queensland, Brisbane, QLD 4072, Australia; r.vinarao@uq.edu.au (R.V.); c.proud@uq.edu.au (C.P.); s.fukai@uq.edu.au (S.F.); 2 Department of Primary Industries, Yanco Agricultural Institute, Yanco, NSW 2703, Australia; peter.snell@dpi.nsw.gov.au

**Keywords:** QTL validation, KASP, molecular markers, aerobic production, rice, root angle

## Abstract

Aerobic rice production (AP) provides potential solutions to the global water crisis by consuming less water than traditional permanent water culture. Narrow root cone angle (RCA), development of deeper rooting and associated genomic regions are key for AP adaptation. However, their usefulness depends on validation across genetic backgrounds and development of linked markers. Using three F_2_ populations derived from IRAT109, *qRCA4* was shown to be effective in multiple backgrounds, explaining 9.3–17.3% of the genotypic variation and introgression of the favourable allele resulted in 11.7–15.1° narrower RCA. Novel kompetitive allele specific PCR (KASP) markers were developed targeting narrow RCA and revealed robust quality metrics. Candidate genes related with plant response to abiotic stress and root development were identified along with 178 potential donors across rice subpopulations. This study validated *qRCA4*’s effect in multiple genetic backgrounds further strengthening its value in rice improvement for AP adaptation. Furthermore, the development of novel KASP markers ensured the opportunity for its seamless introgression across pertinent breeding programs. This work provides the tools and opportunity to accelerate development of genotypes with narrow RCA through marker assisted selection in breeding programs targeting AP, which may ultimately contribute to more sustainable rice production where water availability is limited.

## 1. Introduction

To feed the increasing number of rice consumers by 2030, world rice production has to increase by 40% [1]. This is a tall order considering the devastating negative effects of climate change such as looming water crisis brought about by climate change, increasing population, and higher food demand [2]. Aerobic rice production (AP) system is a promising technology which may provide solutions to this conundrum. AP system is an intensive rice farming method consisting of direct seeded rice cultivation under non-flooded conditions and is usually well-irrigated [3]. Compared with upland cultivation, AP has higher input levels and does not encounter drought [4]. Although water availability in AP system is generally high, transient water deficit may develop between irrigation events. This is distinct from water deficit as commonly perceived in relatively low yielding upland cultivation.

Several physiological and morphological attributes have been postulated to minimise effect of intermittent water deficit and enable stable or increased rice yield in AP systems. These include (1) vigorous biomass production with harvest index ranging from 0.40–0.48, (2) improved yield component traits such as increased number of spikelets per unit area, (3) plants with intermediate height (< 130 cm), larger and thicker tillers and leaves, and (4) improved root system architecture (RSA) such as increased rooting depth [4]. RSA is the arrangement of the crop root system in terms of specific geometric configuration across a rooting medium, and is particularly important as it determines anchorage, soil nutrient and water exploitation, and developmental plasticity, which will ultimately affect maximum yield and yield stability. One of the components of RSA is rooting angle-root cone angle (RCA), is the plant’s root angle relative to the vertical axis. Genotypes with narrow RCA tend to have deeper rooting systems [5] and is therefore highly relevant for AP adaptation. Several experiments in upland field conditions with drought stress have shown the advantage given by narrow RCA. Using 12 cultivars, it was shown that narrow RCA was associated with development of deeper roots [6]. Similarly, genotypes with higher ratio of deep roots (RDR, narrow RCA) tended to have increased rooting depth in an upland field condition with drought stress [5]. The advantage of having deeper roots in an AP system was indicated with positive genetic correlations observed between percentage of deep roots and grain yield when a set of 20 diverse cultivars were evaluated [7]. To facilitate the development of genotypes with narrower RCA and deeper rooting and their further inclusion in breeding programs, identification of genes and quantitative trait loci (QTL) governing these traits is imperative.

To date, a number of studies conducted in paddy fields or in transplanted systems identified genomic regions associated with rooting angle. Using Kinandang Patong, several deep rooting QTL (*DRO1, DRO2, DRO3, DRO4* and *DRO5*) located in different chromosomes were identified [5,8,9,10]. Of these, only *DRO1* was cloned and was shown to increase rooting depth of genotypes with favourable allele, thereby stabilising rice yield in upland conditions and under drought stress. IRAT109, an upland tropical japonica (tropjap) genotype, was also utilised to map QTL for RDR in transplanted systems, identifying *qRDR-2* as a genomic region located in chromosome 2 [11]. Recently, novel, stable, consistently identified across environments and environment-specific genomic regions conferring narrow RCA were identified in AP systems [12]. *qRCA4* was found to be a major and stable QTL identified across all environments tested, and *qRCA1.1* was a novel QTL which was also expressed in all environments. Of the environment specific QTL, *qRCA2.1* and *qRCA2.2* were found to be associated in intermittent water stress conditions (IWS) and was shown to have moderate to high effects. In order to facilitate the introgression of these QTL in relevant breeding programs, it is imperative to demonstrate the effect of these genomic regions in multiple genetic backgrounds [13]. Additionally, molecular markers tagging these QTL, and ultimately the target trait-narrow RCA and deeper roots will also be vital for seamless introgression of these loci into breeding programs.

A number of molecular markers have been developed and used extensively in rice improvement efforts [14,15] including restriction fragment length polymorphism, random amplified polymorphic DNA, cleaved-amplified polymorphic sequence, simple sequence repeat (SSR), and single nucleotide polymorphism (SNP) markers. Numerous SSR markers were developed by McCouch, et al. [16] and their corresponding sequences are available online (https://archive.gramene.org/markers/, accessed on 18 June 2021). SSRs are most widely used markers in major cereals crops as they are highly reproducible, co-dominant, relatively simple and cheap to use, and generally highly polymorphic. SSRs do come with some disadvantages such that they generally give information to a single locus only per assay, have difficulty in merging data across platforms and cumbersome curation of allele sizes into databases. With the advent of whole genome sequence information, SNPs became the marker of choice for majority of high throughput genotyping applications because of (1) they are ubiquitous in eukaryotic genomes, (2) cost effectiveness in terms of assay and automation, and (3) simple allele calling, data analysis and curation into databases due to their bi-allelic nature [17]. Recently, high-density SNP chips called the Cornell-IR RiceLD Array have been developed at the International Rice Research Institute (IRRI) in collaboration with Cornell University (https://isl.irri.org/services/genotyping/7k, accessed on 18 June 2021). This chip allows for the simultaneous genotyping of a sample across 7000 SNP loci including 21 gene-based SNP diagnostic for submergence tolerance *SUB1A* gene, grain quality characteristics, and loci for resistance to bacterial leaf blight, blast, brown planthopper and tungro. One disadvantage of this SNP chip is that it is fixed (SNP markers and traits), similar to an amplicon-based SNP genotyping assay developed recently [18]. On the other hand, there are a number of available high-throughput technologies able to run flexible sets of SNP markers. Kompetitive allele-specific polymerase chain reaction (KASP) marker assay is a single-step genotyping technology that uses two competing allele-specific forward primers, one reverse primer, and a master mix with a fluorescence resonance energy transfer cassette and *Taq* DNA polymerase. With this, pre-identified, co-dominant alleles for SNPs and insertion/deletion variants between parents, progeny and other genotypes being tested are determined [19]. The Genotyping Services lab of IRRI have developed and validated trait based KASP SNP markers for the following: yield components (grain number, grain size, and panicle architecture), grain quality (amylose, chalk, and gelatinization temperature), abiotic stress tolerance (anaerobic germination, drought, cold, and submergence), and disease and pest resistance (bacterial blight, blast, brown planthopper, and tungro) (https://isl.irri.org/services/genotyping/trait-based-genotyping, accessed on 18 June 2021). To our knowledge, KASP SNP markers targeting root angle, whether in conventional flooded or AP systems, have not been developed and validated to date. Using genotypes with known *qRCA* composition and newly developed segregating populations, this study was carried out to validate the effect of *qRCA* QTL across several genetic backgrounds and to develop KASP-based SNP markers targeting *qRCA* QTL for its precise introgression into various genetic backgrounds pertinent to rice breeding programs, particularly targeting environments exposed to transient water deficit. Additionally, this study also determined molecular marker quality metrics of the newly developed markers and identified potential donors using publicly available databases.

## 2. Results

### 2.1. Phenotypic Variation of the Segregating Populations

Highly significant variation (*p* < 0.001) for RCA was observed in all three F_2_ populations across each of the three measurement times, with 42 days after sowing (DAS) showing the highest heritability ranging from 0.67 to 0.69 (Table 1). Highly significant positive correlations were also observed between measurement times in the three populations for IRAT109/Norin PL8 (IRNO) (*r* = 0.61–0.82 **), IRAT109/Langi (IRLA) (*r* = 0.68–0.87 **), and IRAT109/RL11 (IRRL) (*r* = 0.76–0.89 **) populations, respectively with the highest correlation between RCA measured at 42 and 49 DAS for each population. With these results across the populations tested and the relatively high heritability, the data collected from 42 DAS were used in subsequent analyses.

Examining the phenotypic data of the populations, IRRL showed the largest range in terms of RCA, followed by IRLA, and IRNO with the smallest range (Table 1). In terms of the population mean, a similar trend was also observed with IRRL which had a mean RCA of 98°, followed by IRLA with 90°, and then IRNO with 85°. Significant genotypic variation was also observed in the checks included in the evaluation. Of the checks, IRAT109 produced the narrowest RCA ranging from 71 to 79°. Norin PL8 tended to have relatively narrow RCA ranging from 93 to 98°. All the other checks (Langi, Reiziq, RL11, and Sherpa) included in the experiments showed relatively wide RCA, ranging from 102 to 111°, 117 to 124°, 95 to 122°, and 101 to 117°, respectively.

### 2.2. SNP Selection Using 3KRGP

A total of 40 SNPs unique to IRAT109 were identified across the *qRCA4* region ±100 kb of the confidence interval. Initially, all 40 SNPs showed that the average IRAT109 SNP allele frequency was 44.7%, 33.9%, 3.6% and 13.6% in tropical japonica (tropjap), subtropical japonica (subtrop), temperate japonica (tempjap), and all3K Rice Genotyping Project (3KRGP, all3K), respectively. A linkage disequilibrium (LD) map was constructed using these 40 SNPs and based on the Tajima D’ values, the *qRCA4* region was found to have three linkage blocks whether the analysis was carried out using the japonica subgroup or all3K genotypes (Figure S1). This was indicative that a total of three SNP markers were required to track the introgression of the whole *qRCA4* region. After filtering for low allele frequencies, a total of 12 SNPs were retained with an average allele frequency of 24.6%, 5.4%, 1.5% and 3.7% in tropjap, subtrop, tempjap, and all3K, respectively. Marker development for *qRCA4* was prioritised since it was shown to be a major genomic region associated with narrow RCA, although SNP markers for other genomic regions were also developed. For *qRCA1.1*, *qRCA2.1*, and *qRCA2.2*, two, one and one SNP were selected near the peak marker previously reported, respectively. The strategy for selection of SNP for *qRCA2.2* was similar to that of *qRCA4*, while it was a bit different for *qRCA1.1* and *qRCA2.1,* where SNPs selected had moderate allele frequencies in tempjap since the favourable allele for these loci came from Sherpa, a tempjap genotype. In total, a set of 16 SNPs were selected and subsequent KASP marker assays were developed targeting *qRCA1.1*, *qRCA2.1*, *qRCA2.2* and *qRCA4.*

### 2.3. Molecular Marker Development and Quality Metrics of KASP Markers Targeting qRCA QTL

SNP genotype information from the validation plate was processed and visualised to identify well-performing markers and cull-out poor performing ones. Upon visual inspection of the 16 markers, two markers—snpOS00936 and snpOS00943 were immediately discarded as they were monomorphic and unable to call heterozygotes, respectively (Figure S2). On the other hand, molecular markers snpOS00939 and snpOS00948 were polymorphic but showed close clustering among alleles and further inspection was carried out. All other markers showed acceptable clustering and were considered for further analysis. Using the known QTL composition of 54 recombinant inbred lines (RILs) included in the validation plate, false positive rate (FPR) and false negative rate (FNR) were computed per molecular marker. From this analysis, two molecular markers were further discarded as they showed high FPR. The SNP marker snpOS00939, which also showed close clustering, had an FPR of 26.1% and an FNR of 6.5%. Another discarded marker was snpOS00946, which initially showed good clustering, but further analysis showed a very high FPR of 54.5% and an FNR of 3.1%. In total 12 markers showed acceptable FPR and FNR and were considered good candidates to validate the effect of QTL mentioned above across multiple genetic backgrounds (Table 2). On average, the newly developed KASP-based SNP molecular markers showed excellent call rates of 99.56% and ranged from 97.87–100.00%. FNR was also relatively low with an average of 2.27%, ranging from 0.0 to 8.3%. Finally, FPR ranged from 3.3 to 9.5%, with an average of 7.61%. In summary, nine markers (snpOS00933, snpOS00934, snpOS00935, snpOS00937, snpOS00938, snpOS00940, snpOS00941, snpOS00942 and snpOS00944) targeting *qRCA4* and one each for *qRCA1.1* (snpOS00945), *qRCA2.1* (snpOS00947), and *qRCA2.2* (snpOS00948) were used to validate their respective effects across genetic backgrounds. For *qRCA4*, the nine markers were distributed across the previously reported confidence interval of the QTL. Two of the markers, snpOS00933 and snpOS00944 were designed to be flanking the QTL, while the other seven were designed within the QTL. On average, the physical inter-marker distance was 136.7 kilobase (kb), with the highest distance (273.3 kb) observed between snpOS00933 and snpOS00934 and the lowest (41.2 kb) between snpOS00940 and snpOS00941.

Examining the genotype composition of the six varieties, the nine markers targeting *qRCA4* were shown to be polymorphic between the donor IRAT109, and other genotypes—Langi, Reiziq, RL11 and Sherpa. In the case of Norin PL8, of the nine markers, six were shown to be polymorphic, while the other three (snpOS00934, snpOS00938, and snpOS00941) were monomorphic. For *qRCA1.1*, Reiziq and RL11, including IRAT109, showed unfavourable allele, while for *qRCA2.1*, only IRAT109 showed unfavourable allele. Lastly, for *qRCA2.2*, except for IRAT109, all genotypes have unfavourable allele.

### 2.4. QTL Validation and Effects across Genetic Backgrounds

Three F_2_ populations were used to validate the effects of *qRCA* QTL in different genetic backgrounds. Of the nine *qRCA4* markers developed, two (snpOS00937 and snpOS00942) were excluded from the analysis as they were calling IRAT109 as heterozygote. Downstream analysis was carried out using the remaining seven markers. Across the three populations, all the seven markers followed the expected Mendelian segregation ratio of F_2_ (χ^2^_[1:2:1]_ = 0.57–4.86, *p* > 0.05) populations (Table S1). This indicated conformance of both the molecular makers and the populations with expected ratios. Estimation of the genetic distance between the markers revealed a total of 5.70 centiMorgan (cM) distance between the two flanking markers, with an average distance of 0.97 cM between markers. Evaluation of the effect of *qRCA4* across the three populations using these markers revealed significant associations in two populations: IRLA and IRRL, while there was no significant association detected in the IRNO population (Table 3). Examining the individual populations, significant association was revealed in IRRL population with logarithm of odds (LOD) of 13.36. Introgression of the favourable IRAT109 allele (BB) suggested an effect of narrower RCA of about 15.12°. Additionally, a significant dominance effect was also detected, indicating the heterozygotes have 4.08° narrower RCA than expected, based on the estimated additive effects of the IRAT109 allele. In this population, using these molecular markers, *qRCA4* was also shown to explain 17.27% of the genotypic variation present in IRRL. Furthermore, the effect of *qRCA4* in IRLA had a LOD of 4.13 and the introgression of the IRAT109 allele showed an estimated effect of 11.87° narrower RCA, compared with that of the unfavourable allele (AA). A significant dominance effect was also detected, with heterozygotes having 1.02° narrower RCA and the region also explained 9.25% of the variation present in IRLA population. In terms of the third population, no significant association was detected, indicating that the other parent in this population (Norin PL8) may already possess the *qRCA4* locus, which is further supported by the presence of three monomorphic markers between the parents and is also congruent with the phenotypic observations for Norin PL8’s RCA carried out above.

**Figure 1:**
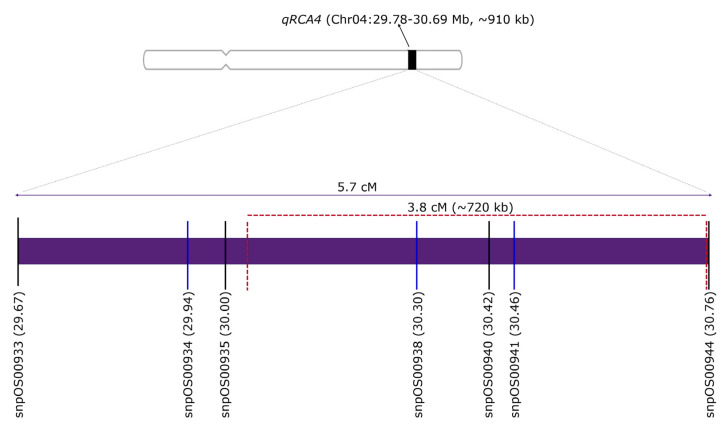
Molecular map of *qRCA4* using newly designed molecular markers and validation results from two populations. Black lines indicate polymorphic markers between IRAT109 and other check genotypes used, while blue lines indicate monomorphic markers between IRAT109 and Norin PL8. Red dashed lines indicate the new confidence interval computed.

Bayesian credible confidence intervals were also estimated using the association detected in the two populations above. Using the IRRL population, the *qRCA4* confidence interval was delimited between 1.13 and 5.70 cM, translating between 29.88 and 30.76 megabase (Mb) physical position of chromosome 4. For IRLA population, *qRCA4* was delimited between 1.92 and 5.70 cM (30.04–30.76 Mb). Combining these two results, it was shown that *qRCA4* was mapped into ~720 kb region of chromosome 4 (Figure 1). Compared with previous results, we can now confidently indicate that this valuable genetic region is flanked between snpOS00935 and snpOS00944.

In terms of the validation of effects of *qRCA1.1*, *qRCA2.1*, and *qRCA2.2*, SNP markers snpOS00945, snpOS00947, and snpOS00948 were used, respectively. It was noted that for IRNO, snpOS00947 did not follow expected Mendelian segregation ratio for (χ^2^_[1:2:1]_ = 39.13, *p* < 0.05) and for IRLA, both snpOS00945 and snpOS00947 also did not follow expected ratios (χ^2^_[1:2:1]_ = 7.08–16.35, *p* < 0.05). On the other hand, snpOS00948 followed the expected F_2_ ratio (χ^2^_[1:2:1]_ = 0.21–1.88, *p* > 0.05). Unfortunately, single marker analysis (SMA) carried out using these markers indicated no significant effect in the RCA across three populations tested. With these results, no downstream analyses were carried out for these loci and SNP markers.

### 2.5. Performance of Newly Developed KASP Markers Targeting qRCA4 Locus

**Figure 2:**
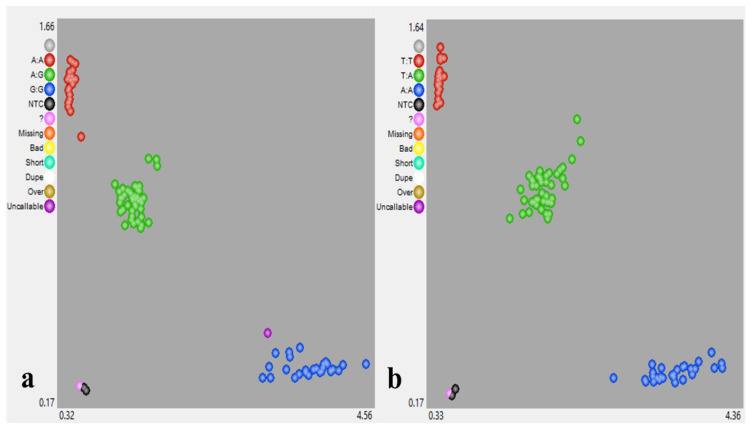
Cluster genotype of KASP molecular markers (**a**) snpOS00938 and (**b**) snpOS00941 using F_2_ segregating population.

Utilising the F_2_ SNP genotyping data, molecular marker quality metrics were analysed for the seven markers. On average, the *qRCA4* targeting markers showed a call rate of 99.21%, with snpOS00940 and snpOS00944 showing the highest call rates at 99.51%, while snpOS00933 had 98.64% (Figure 2). An additional 13 genotypes important to the Australian breeding program were also genotyped for *qRCA4* markers. This analysis showed three genotypes (55 A, Moroberekan, and YRF210) possess the *qRCA4* favourable alleles. Bringing these results together with the checks previously genotyped, it can be noted that the breeding program specific utility of these markers was at about 83%.

### 2.6. Identification of Candidate Genes and Possible Donors qRCA4 in 3K in All Subpopulations

Through the 3KRGP, potential donors for *qRCA4* were identified among different rice subpopulations. Since the original donor, IRAT109, is a tropjap genotype, it was expected that there were more donors in this subpopulation. Indeed, 157 out of 372 (42.2%) tropical japonicas possessed the favourable alleles of *qRCA4*. In terms of the other subpopulations, a total of 21 potential donors were identified (Table S2). A single donor, Y134, from the indica subpopulation was identified while two, IAS 22-8 Palmar and CX578, were identified for the tempjap subpopulation. The remaining 18 were representatives of the subtrop population, majority of which (13) originated from Lao People’s Democratic Republic, two from Thailand, and a single accession from China.

Examining the annotated genes within the *qRCA4* region, it was found that there were 111 genes with transcript evidence, with 22 of them having alternative transcripts or splice variants. Of the 111 genes, 83 have been identified to have known molecular and/or biological functions or have been shown to have high similarity with known proteins, while the other 28 code for hypothetical genes or non-protein coding transcript. Of those identified with known functions, four have been identified to be associated with plant response to abiotic stress stimulus (LOC_Os04g50820, LOC_Os04g50880, LOC_Os04g50990, and LOC_Os04g51330). The SNP marker, snpOS00938 sits within the sequence of LOC_Os04g51172, and is predicted to be an intron variant of the gene. Additionally, it was noted that snpOS00941 is a downstream gene variant of LOC_Os04g51440, a putative villin protein and have been shown to be important regulator of actin. It is orthologous to the *Arabidopsis* gene AT4G30160 with a putative villin 4 function.

## 3. Discussion

### 3.1. qRCA4 Is Effective across Multiple Genetic Backgrounds

Due to variation in genetic backgrounds, a genomic region identified to be effective in one mapping population may not necessarily be identified in other genetic backgrounds. Validation of the effect of these QTL in multiple genetic background is necessary before more downstream work can be carried out i.e., gene cloning and integration in breeding programs. This study utilised F_2_ segregating populations derived from three different genetic backgrounds to validate the effect of a major genomic region, *qRCA4*, controlling narrow root cone angle in AP systems. This region was shown to have significant effects in two (RL11 and Langi) out of three genetic backgrounds, with effects ranging from a decrease of 11.7°–15.1° in RCA, explaining between 9.3 and17.3% of the genetic variation present, further strengthening its significance and utility in improving rice AP adaptation. In rice, similar strategies have been carried out, such as the case of *SUB1A* QTL mapping and molecular breeding. *SUB1* was first mapped using F_3_s derived from IR40931-26/PI543581 [20], and then several independent studies confirmed this QTL across several genetic backgrounds [21,22]. These laid down the foundation towards breeding for Sub1 mega-varieties through the subsequent identification of causative gene and SNP molecular markers which enabled transfer through marker assisted selection (MAS) [23]. Following this, validation of *qRCA4* is therefore a critical step not only to ensure its effectiveness across several genetic backgrounds but to also warrant its utility in breeding programs through MAS. Similar to the present results, *DRO2* was also detected in three genetic backgrounds, ARC5955, Pinulupot 1, and Tupa729, and was shown to be related with deeper rooting in transplanted systems [8]. In the population derived from Norin PL8, no significant association was detected although significant phenotypic variation existed in the F_2_ indicating that other genomic regions may play a role in the control of RCA in this population. It can also be noted that three markers utilised in this study were monomorphic between IRAT109 and Norin PL8, in addition to Norin PL8 showing narrower RCA compared with the other checks evaluated in this study. This further supports the notion that Norin PL8 already possess the *qRCA4* locus.

### 3.2. KASP-Based SNP Markers Tagging qRCA4 Were Developed

Molecular markers are essential for the seamless introgression of target traits into recipient genotypes carried out through MAS in a typical molecular breeding program. In rice, a suite of molecular markers has been designed and developed tagging high value traits such as yield components, grain quality, and disease resistance [24,25]. This study, for the first time, reports the simultaneous validation and development of KASP based SNP markers targeting *qRCA4*, a highly valuable genomic region for AP adaptation. With the advent of more cost-effective whole genome sequencing technologies, SNPs became the marker of choice in many breeding programs. Using the Fluidigm SNP genotyping platform, Kim, et al. [26] developed SNP markers targeting yield enhancing loci—*Gn1a*, *SPL14*, *Ghd7*, *GS5* and *GS3*. Gel-based SNP markers have also been developed targeting blast and bacterial blight resistance genes, *Pita* and *xa5*, respectively [27]. These other SNP genotyping platforms were also available but KASP has been in the forefront of these as it is more preferred due to its cost effectivity, assay conversion rate, accuracy and flexibility [19], especially in the context of marker assisted backcrossing and marker assisted recurrent selection applications. KASP technology has also been shown to work using single seed genotyping in rice, further facilitating its adoption in breeding programs by allowing breeders to combine rapid generation advance techniques more effectively [28]. To our knowledge, these are the first high throughput SNP markers targeting root growth angle traits developed, whether in traditional flooded or in AP systems. KASP markers have been developed at IRRI, but have not included root traits such as RCA. An independent work by Steele, Quinton-Tulloch, Amgai, Dhakal, Khatiwada, Vyas, Heine and Witcombe [24] also developed KASP markers but only included disease resistance and grain quality traits. Additionally, Yang, Chen, Chen, Sun, Huang, Zhou, Huang, Wang, Liu, Wang, Chen and Guo [25] also developed a core KASP SNP set tailored for rice varietal assessment, genetic diversity analysis, and also included markers for agronomic traits such as yield, quality, resistance, fertility, and phenology.

Using the newly developed KASP markers, this present study also delimited the confidence interval of the *qRCA4* QTL into ~720 kb region. This confidence interval is flanked by markers snpOS00935 and snpOS00944. Additionally, SNP markers snpOS00938 and snpOS00941, may also be the most useful markers as they may represent the variation that is indicative of the presence of *qRCA4*. In terms of *qRCA1.1*, *qRCA2.1* and *qRCA2.2*, no markers were developed associated with these as the markers used did not show significant association with RCA in the tested populations. This may be due to several reasons. First, the markers tested were very limited, only one each for the individual QTL and may warrant the design of additional markers to saturate each region. Second is that these QTL might be background specific which further highlights the importance of this QTL validation exercise. Lastly, in terms of *qRCA2.1* and *qRCA2.2*, the markers targeting them may have not shown effect since these QTL were only shown to be significant in IWS conditions [12]. Further testing of the designed markers in IWS conditions should be carried out to definitively check the association of these markers with RCA.

Examining the LD map for *qRCA4*, the four markers identified above represent the three linkage groups detected and are therefore enough to ascertain the introgression of the *qRCA4* locus. Molecular marker quality metrics (as described by Platten, et al. [29]) of these four markers also showed high call rates, low FNR, and relatively low FPR. By genotyping lines relevant to the Australian breeding program, it has also shown the predicted high utility of these markers. The KASP assay, compared with other fixed SNP arrays, are flexible and individual markers can be run together with other markers. By developing *qRCA4* markers in KASP system, this also ensures its seamless integration in breeding programs and can be easily pyramided with other high-value AP adaptation genes/QTL such as those for cold tolerance.

### 3.3. 3KRGP and Database Search Facilitated the Identification of Potential Donors and Candidate Genes

In any breeding program, the availability of donor and favourable alleles for traits of interest is very important. In cases where these alleles/traits are not available, exotic germplasm such as wild progenitors may be used but this would take an arduous effort to transfer the traits into cultivated germplasm. It is therefore highly important to identify readily available donor genotypes for use in specific breeding programs. Using the publicly available 3KRGP SNP genotyping data [30], allele mining was carried out to identify possible donors within specific subpopulations. Donors in various subpopulations were identified including indica, tempjap, tropjap and subtrop. These donors are available at IRRI and may be requested if deemed useful for the particular program. This study also highlights the utility of publicly available databases to augment in discovery process and help breeders make more informed decisions, as in this case, donor parent selection.

Using the newly determined confidence interval, candidate genes along the *qRCA4* region were also identified through RAPDB [31]. Genes related with plant response to abiotic stress stimulus were identified, including LOC_Os04g51330. LOC_Os04g51330 was similar to a maltose excess protein, a probable maltose transporter vital for the conversion of starch to sucrose in leaves at night [32] and more importantly, an orthologue of the *Arabidopsis* root cap protein 1. On the other hand, LOC_Os04g51440, was identified which is an orthologue of to the *Arabidopsis* gene AT4G30160 (putative VILLIN 4). *In Arabidopsis*, VILLIN 4 proteins have been to be involved in root hair growth through the regulation of actin organisation [33], and in rice, VILLIN 2 was shown to be important in regulating plant architecture through the modulation of microfilament dynamics and polar auxin transport [34]. These genes represent excellent targets for downstream cloning experiments to characterise the molecular function of the gene associated with *qRCA4* and narrow RCA in AP systems. Emerging technologies for gene cloning such as CRISPR (Clustered Regularly Interspaced Short Palindromic Repeats) with CRISPR-associated protein Cas9 (CRISPR-Cas9) holds promise and may be utilised to precisely engineer and target variations present in the *qRCA4* region and candidate genes of interest [35]. Non-targeted metabolic profiling of genotypes with contrasting RCA phenotypes and *qRCA* composition across several environmental conditions may also reveal biochemical pathways and specific metabolites which may aid in understanding mechanisms related with rice plant’s ability to cope with and tolerate stress involving transient water deficit. Finally, transcriptomic profiling and network analysis of contrasting genotypes may show differentially expressed genes in modules and along with metabolic profiling, may provide a systems level understanding of rice response to transient water deficit, and ultimately, AP adaptation.

## 4. Materials and Methods

### 4.1. Plant Materials

To validate the effect of target QTL in different genetic backgrounds, progenies were produced between IRAT109 and three recipient parents (RP), RL11, Langi, and Norin PL8. F_1_s were produced by crossing IRAT109 with RPs, and subsequently, F_2_ progenies were produced by selfing F_1_s. A total of 810 F_2_ progenies were genotyped and evaluated for RCA. Of these, 283, 196 and 331 were derived from crosses between IRNO, IRLA, and IRRL, respectively. A total of 60 genotypes were utilised to test the molecular marker quality metrics of the newly developed markers. Of these, six were either released varieties or donors of high importance to the Australian breeding program—IRAT109, Sherpa, Reiziq, Langi, RL11 and Norin PL8. The other 54 genotypes were RILs derived from Sherpa/IRAT109 and were previously identified to have varying combinations of QTL for RCA [12].

### 4.2. Phenotyping for RCA

Three experiments were carried out to evaluate the segregating populations developed above, along with the six varieties/checks following the clear pot method described by Vinarao, Proud, Zhang, Snell, Fukai and Mitchell [12], with some modifications, at the University of Queensland (UQ) St Lucia (27.4975° S, 153.0137° E) campus. Briefly, single seeds of F_2_s and check genotypes were direct seeded in 4 L clear pots (ANOVApot^®^, 200 mm diameter, 190 mm height, http://www.anovapot.com/php/anovapot.php, accessed on 18 June 2021) filled with pine bark potting media (70% composted pine bark 0–5 mm, 30% coco peat, pH 6.35, EC = 650 ppm, nitrate = 0, ammonia < 6 ppm and phosphorus = 50 ppm) with 3 g/L Osmocote Exact 3–4M (19-9-10 + 2MgO + TE, ICL Specialty Fertilizers), 2 g/L Osmocote Exact 5–6 M (15-9-12 + 2MgO + TE) and 0.82 g/L Suscon Maxi Green (Nufarm, Melbourne, VIC, Australia). Using a pair of forceps, seeds were sown vertically at a depth of 3 cm along the pot wall with a final density of 12 plants per pot. In every experiment, parental genotypes for each specific F_2_ population were replicated in every pot, while the other check genotypes were replicated every incomplete block (four clear pots/incomplete block). To prevent penetration of light, similar sized black pots were used to cover the clear pots. Pots were placed on a table and a constant layer of water (~4 cm from the base of the inner pot) was maintained to mimic aerobic growing conditions. The experiments were carried out in a temperature-controlled glasshouse set at 28 °C/21 °C day/night temperatures with natural light. RCA were measured manually at 35, 42 and 49 DAS using a protractor, by measuring the cone angle between the two most external nodal roots.

### 4.3. Development of KASP SNP Molecular Markers

SNP variations across target QTL regions were investigated using the 3KRGP database which included IRAT109, the main donor genotype in this study as well as six varieties relevant to the Australian breeding program (M7, M102, M401, M203, Calrose 76 and YRM6-2). For *qRCA4*, SNPs unique to IRAT109 were identified along the region, ±100 kb upstream and downstream the QTL’s confidence interval. Using the SNPs identified, LD in the region was investigated using TASSEL [36] by computing the standardised disequilibrium coefficient (D’) from the full matrix, in both the japonica sub-group and all the genotypes included in 3KRGP. Additionally, allele frequencies of the selected SNPs were also determined using the SNP-Seek database [30]. Their relative allele frequencies were noted in different populations: tropjap, subtrop, tempjap and in all3K. SNPs having the desired allele frequencies were selected and analysed further. DNA sequences surrounding the SNP of interest (±200 bp) were downloaded from the SNP-Seek database and multiple sequence alignment was carried out using CLUSTALW implemented in Molecular Evolutionary Genetic Analysis X [37] software to ascertain that the region surrounding the target SNP was fairly conserved or with minimal variation. Finally, the downloaded IRAT109 sequence was aligned through basic local alignment search tool with the Nipponbare sequence implemented in the Rice Annotation Project Database (RAP-DB) to confirm that the regions selected were unique and only occurred in the target chromosome. SNPs and their surrounding sequences which passed the criteria described above were compiled and sent to Intertek, Australia (https://www.intertek.com/agriculture/agritech/, accessed on 18 June 2021) for subsequent development of KASP molecular markers. To constitute a plate to validate newly designed molecular markers, IRAT109 and Sherpa were replicated four times while Reiziq, Langi, RL11, and Norin PL8 were replicated thrice. Leaf samples of IRAT109 were also combined with other genotypes (Sherpa, Reiziq, Langi, RL11, and Norin PL8) to create heterozygotes (a total of 20 samples). To complete the set, leaf samples of the 54 RILs derived from IRAT109/Sherpa were also included, bringing the total to 94. Two 4–6 mm leaf samples were sampled and placed per well of an Abgene^TM^ 96 deepwell storage plate (ThermoFisher Scientific, Waltham, MA, USA) to constitute the validation plate. Leaf samples were subsequently freeze-dried at −55 °C for 48 h using Alpha 1–2 LDplus (Martin Christ, Osterode am Harz, Germany) and then sealed using 96-well sealing mats (ThermoFisher Scientific, Waltham, MA, USA). Upon selection of well-performing markers, a similar method was used to prepare the F_2_ segregating genotypes. KASP genotyping of validation genotypes and segregating population was carried out at the Intertek, Australia laboratory following standard manufacturer specifications.

### 4.4. Molecular Marker and QTL Validation

Cluster graph for each SNP genotyping assay was visualised using KlusterCaller^TM^ and SNPviewer software (LGC Biosearch Technologies, Teddington, UK). In some instances where automated SNP genotype calling of the software classified some individuals into ‘uncallable’ but were clearly belonging into a cluster, these were manually called into their respective cluster genotypes. Newly developed KASP molecular markers were evaluated for core quality metrics as described by Platten, Cobb and Zantua [29], which included call rate, FPR, FNR and utility. FPR was the proportion (percent, %) of known genotypes (recipients) which were incorrectly classified as having the QTL, and was computed with the following equation:

$$\mathrm{FPR}\;\left(\%\right)=\frac{\#\;of\;recipients\;with\;favourable\;allele\;}{Total\;\#\;of\;known\;recipients}\;\times \;100$$

On the other hand, FNR was the proportion of donor genotypes (known to harbour QTL) which were incorrectly classified to not possess the QTL due to not having the favourable allele of a marker, and was given by the following equation:

$$\mathrm{FNR}\;\left(\%\right)=\frac{\#\;of\;donors\;with\;unfavourable\;allele\;}{Total\;\#\;of\;known\;donors}\;\times \;100$$

To estimate these molecular marker metrics, a set of 60 genotypes identical to the genotypes specified above were assembled and genotyped using the newly designed KASP markers targeting *qRCA4*, *qRCA1.1*, *qRCA2.1* and *qRCA2.2*. Of the 54 RILs, 23, 22, 21 and 30 of them had *qRCA4*, *qRCA1.1*, *qRCA2.1* and *qRCA2.2*, respectively. Based on the QTL composition of the RILs, FPR and FNR of the individual markers were analysed and were subsequently used to select markers used to genotype the segregating F_2_ populations for succeeding QTL validation.

Three F_2_ populations: IRLA, IRNO and IRRL were genotyped with selected markers to validate the effect of *qRCA* QTL across genetic backgrounds. Composite interval mapping was carried out to validate the effect of *qRCA4* across multiple genetic backgrounds, and then stepwiseqtl, refineqtl and fitqtl functions in R/qtl were used to estimate the QTL effect [38]. Bayesian credible confidence interval was determined using bayesint function of R/qtl. SMA was carried out in R [39] to test for the association of marker with RCA in case of *qRCA1.1, qRCA2.1* and *qRCA2.2* and R^2^ was computed to estimate the proportion of phenotypic variation contributed by individual markers to the phenotype.

### 4.5. Identification of Candidate Genes and Possible Donors Present in 3KRGP

Using the new confidence interval calculated for *qRCA4*, details of the annotated genes located within the region were batch downloaded from RAP-DB which included information such as gene ID, gene function, strand, physical position and gene ontology [31]. Possible candidate genes controlling *qRCA4*-mediated reduction in RCA were identified based on the gene function along with ontology, giving priority with those involved in root development as well as those related with response to abiotic stress stimulus.

Allele frequencies of the candidate SNP molecular markers were investigated using the 3KRGP data available online [30]. Genotypes possessing the favourable *qRCA4* allele were identified in each subpopulation which may represent donors for breeding programs dealing with specific subpopulations.

### 4.6. Statistical Analysis

A mixed linear model was carried out for the analysis of the phenotype data per population which was executed in ASReml (V4.1; VSNi, UK) package running in the R environment [39]. Parents of the respective F_2_ population and other checks were treated as fixed effects while the F_2_ plants were treated as random effects. Best linear unbiased predictors were then calculated after accounting for the randomisation and other blocking parameters. Heritability for each experiment (F_2_ population) was computed as specified by Smith, et al. [40]. Additionally, chi-square test for deviation from expected Mendelian F_2_ segregation ratios (1:2:1) of the SNP genotype data was carried out across the three populations to show either conformance or deviation with expected ratios.

## 5. Conclusions

This study validated the effect of *qRCA4* in multiple genetic backgrounds further reinforcing its value in improvement of rice genotypes for AP adaptation. Simultaneously, high-throughput KASP SNP molecular markers were also developed to tag this genomic region which will facilitate its seamless introgression into target recipient genotypes especially in breeding programs where AP adaptation traits are important. This study reports for the first time the development of such markers associated with root traits, whether in traditional transplanted flooded or in AP systems. Molecular marker quality metrics also show marker robustness and possible high utility in breeding programs. Using 3KRGP, potential donors across subpopulations were identified representing readily available donors for use of prospective breeding programs. Candidate genes along the *qRCA4* region were also identified and were shown to either be associated with plant response to abiotic stress stimulus or related with root development. Physiological analysis of the relationship of RCA and this locus with grain yield and key traits such as those related to photosynthetic rates and metabolic activities under different environmental conditions will shed some light on mechanisms related to its action and further increase the value of this genomic region and associated molecular markers, specifically in AP systems. This study provides the tools to breeders for the development of genotypes with narrow rooting angle and greater depth, through MAS, ultimately for more sustainable rice production in environments where rice is exposed to transient water deficit.

## Figures and Tables

**Table 1:** RCA (°) statistics of F_2_s derived from crossing IRAT109 with three recipient parents.

	IRLA *n* = 196	IRRL *n* = 331	IRNO *n* = 283
Mean	90	98	85
Min	67	68	63
Max	124	130	116
*p* value	<0.0001	<0.0001	<0.0001
Heritability	0.69	0.67	0.68
	Check Genotypes		
IRAT109	71	79	77
Langi	111	106	102
RL11	ND	122	95
Norin PL8	93	98	94
Sherpa	108	117	101
Reiziq	121	124	117

RCA—root cone angle; IRLA—IRAT109/Langi F_2_; IRRL—IRAT109/RL11 F_2_; IRNO—IRAT109/Norin PL8 F_2_; ND—No Data.

**Table 2:** Molecular marker quality metrics of newly developed KASP-based SNP markers targeting *qRCA* QTL.

SNP ID	Chr	Position	QTL	SNP	Favourable Allele	Call Rate	FPR (%)	FNR (%)
snpOS00933	Chr04	29670314	*qRCA4*	T/A	T	98.94	4.3	6.5
snpOS00934	Chr04	29943687	*qRCA4*	A/C	A	97.87	8.7	3.2
snpOS00935	Chr04	30001385	*qRCA4*	C/T	C	100.00	8.7	0.0
snpOS00937	Chr04	30193259	*qRCA4*	G/C	G	100.00	8.7	0.0
snpOS00938	Chr04	30302635	*qRCA4*	A/G	A	98.94	8.7	0.0
snpOS00940	Chr04	30420667	*qRCA4*	A/C	A	100.00	8.7	0.0
snpOS00941	Chr04	30461857	*qRCA4*	T/A	T	100.00	8.7	0.0
snpOS00942	Chr04	30524467	*qRCA4*	A/G	A	100.00	8.7	0.0
snpOS00944	Chr04	30764226	*qRCA4*	G/A	G	98.94	8.7	0.0
snpOS00945	Chr01	39443793	*qRCA1.1*	G/A	A	100.00	4.5	3.1
snpOS00947	Chr02	27974625	*qRCA2.1*	C/T	T	100.00	9.5	6.1
snpOS00948	Chr02	30450101	*qRCA2.2*	A/T	A	100.00	3.3	8.3

FPR—false positive rate; FNR—false negative rate; KASP—Kompetitive Allele-Specific PCR; SNP—single nucleotide polymorphism; Chr—chromosome; Position—position in base pairs.

**Table 3:** Validation of *qRCA4* effects across different genetic backgrounds using F_2_ populations derived from IRAT109.

Population	N	LOD	AE	DE	R^2^
IRLA	196	4.13	−5.87	−1.02	9.25
IRRL	331	13.63	−7.56	−4.08	17.27
IRNO	283	NS	NS	NS	NS

N—number of F_2_ plants tested; LOD—logarithm of odds; AE—additive effect of the allele from IRAT109; DE—dominance effect of the allele from IRAT109; R^2^—percentage of genotypic variation explained by QTL; NS—not significant.

## Data Availability

Not applicable.

## Supplementary Materials

The following are available online at https://www.mdpi.com/article/10.3390/plants10102099/s1, Figure S1: Linkage disequilibrium (LD) map using 40 SNPs selected based on allele frequencies and polymorphism between IRAT109 and selected genotypes. SNP genotype data from a total of 843 taxa from the japonica subpopulation were used to generate the map using TASSEL software. SNPs in red boxes represent the nine SNPs selected for KASP assay, Figure S2: Cluster genotype plot for the validation plate using newly developed molecular markers targeting *qRCA* loci: (A) snpOS00941, (B) snpOS00936, (C) snpOS00939, and (D) snpOS00943. Each genotype plot consisted of DNA extracted from 54 RILs derived from Sherpa/IRAT109, six selected genotypes, their heterozygotes, and no template controls (NTC), Table S1: Chi-square analysis of the observed and expected Mendelian segregation ratio for unfavourable (AA), favourable (BB), and heterozygote (AB) alleles in the F2 populations (POP), Table S2: Potential donors of *qRCA4* locus identified using SNP genotype data publicly available through the 3K Rice Genotyping Project (3KRGP).
