# Supplementary material for: QTL Validation and Development of SNP-Based High Throughput Molecular Markers Targeting a Genomic Region Conferring Narrow Root Cone Angle in Aerobic Rice Production Systems
Source: Plants (Basel). 2021 Oct 3;10(10):2099. doi: 10.3390/plants10102099 (PMC8537842; doi:10.3390/plants10102099)
Supplement: Supplementary file 1 [file plants-10-02099-s001.zip › Supplementary Tables_Tables S1 and S2.pdf]

Table S1. Chi-square analysis of the observed and expected Mendelian segregation ratio for unfavourable (AA), favourable (BB), and heterozygote (AB) alleles in the $F_2$ populations (POP).

| SNP ID | POP | AA | AB | BB | Chi-Square | P Value |
|---|---|---|---|---|---|---|
| snpOS00933 | IRLA | 50 | 101 | 42 | 1.08 | 0.582 |
| snpOS00934 | IRLA | 49 | 103 | 43 | 0.99 | 0.610 |
| snpOS00935 | IRLA | 49 | 104 | 41 | 1.67 | 0.434 |
| snpOS00938 | IRLA | 44 | 109 | 43 | 2.48 | 0.289 |
| snpOS00940 | IRLA | 44 | 107 | 45 | 1.66 | 0.435 |
| snpOS00941 | IRLA | 44 | 107 | 45 | 1.66 | 0.435 |
| snpOS00944 | IRLA | 44 | 106 | 46 | 1.35 | 0.510 |
| snpOS00945 | IRLA | 47 | 111 | 33 | 7.08 | 0.029* |
| snpOS00947 | IRLA | 46 | 118 | 25 | 16.35 | 0.000* |
| snpOS00948 | IRLA | 54 | 91 | 48 | 1.00 | 0.607 |
| snpOS00933 | IRNO | 65 | 134 | 82 | 2.66 | 0.265 |
| snpOS00935 | IRNO | 66 | 137 | 80 | 1.67 | 0.434 |
| snpOS00940 | IRNO | 65 | 138 | 79 | 1.52 | 0.468 |
| snpOS00944 | IRNO | 66 | 141 | 75 | 0.57 | 0.750 |
| snpOS00945 | IRNO | 70 | 151 | 58 | 2.93 | 0.231 |
| snpOS00947 | IRNO | 70 | 179 | 26 | 39.13 | 0.000* |
| snpOS00948 | IRNO | 61 | 146 | 76 | 1.88 | 0.391 |
| snpOS00933 | IRRL | 87 | 171 | 67 | 3.35 | 0.187 |
| snpOS00934 | IRRL | 89 | 166 | 69 | 2.67 | 0.264 |
| snpOS00935 | IRRL | 89 | 168 | 71 | 2.17 | 0.338 |
| snpOS00938 | IRRL | 92 | 168 | 65 | 4.86 | 0.088 |
| snpOS00940 | IRRL | 91 | 170 | 67 | 3.95 | 0.139 |
| snpOS00941 | IRRL | 91 | 168 | 67 | 3.84 | 0.147 |
| snpOS00944 | IRRL | 89 | 170 | 69 | 2.88 | 0.237 |
| snpOS00947 | IRRL | 74 | 169 | 65 | 3.45 | 0.178 |
| snpOS00948 | IRRL | 77 | 155 | 82 | 0.21 | 0.900 |

*: $P < 0.05$

Table S2. Potential donors of *qRCA4* locus identified using SNP genotype data publicly available through the 3K Rice Genotyping Project (3KRGP).

| **Genotype/Variety** | **3KRGP IRIS ID** | **Country of Origin** | **Chr04:30302635** | **Chr04:30461857** | **Subpopulation** |
|---|---|---|---|---|---|
| Y134 | CX15 | - | A | T | Indica |
| IAS 22-8 PALMAR::IRGC 26058-1 | IRIS 313-10967 | Brazil | A | T | Temperate japonica |
| CX578 | CX578 | - | A | T | Temperate japonica |
| LEUANG GLIANG::IRGC 71271-1 | IRIS 313-9123 | Thailand | A | T | Subtropical japonica |
| NAM ROO::IRGC 66996-1 | IRIS 313-10176 | Thailand | A | T | Subtropical japonica |
| CHA LOY OE::C1 | IRIS 313-7856 | - | A | T | Subtropical japonica |
| KAO SONG HAI::IRGC 61827-1 | IRIS 313-11623 | China | A | T | Subtropical japonica |
| SI NAM PUENG::IRGC 75165-1 | IRIS 313-11926 | Thailand | A | T | Subtropical japonica |
| XIENG KANG::IRGC 82533-1 | IRIS 313-12063 | Lao PDR | A | T | Subtropical japonica |
| THATENG::IRGC 86377-1 | IRIS 313-12134 | Lao PDR | A | T | Subtropical japonica |
| SET::IRGC 92200-1 | IRIS 313-12227 | Lao PDR | A | T | Subtropical japonica |
| A MOUK::IRGC 94474-1 | IRIS 313-12252 | Lao PDR | A | T | Subtropical japonica |
| BAN BONG::IRGC 94565-1 | IRIS 313-12254 | Lao PDR | A | T | Subtropical japonica |
| NYAE::IRGC 95302-1 | IRIS 313-12262 | Lao PDR | A | T | Subtropical japonica |
| TA SENG::IRGC 99176-1 | IRIS 313-12312 | Lao PDR | A | T | Subtropical japonica |
| BAN KEO::IRGC 99953-1 | IRIS 313-12323 | Lao PDR | A | T | Subtropical japonica |
| DENG NYAY::IRGC 106907-1 | IRIS 313-12332 | Lao PDR | A | T | Subtropical japonica |
| HAI NA::IRGC 107117-1 | IRIS 313-12342 | Lao PDR | A | T | Subtropical japonica |
| LAB::IRGC 107468-1 | IRIS 313-12346 | Lao PDR | A | T | Subtropical japonica |
| KHAI DO::IRGC 12903-2 | IRIS 313-10679 | Lao PDR | A | T | Subtropical japonica |
| KHAO TAM::IRGC 30085-2 | IRIS 313-11093 | Lao PDR | A | T | Subtropical japonica |
