# Supplementary figures and images for: QTL Validation and Development of SNP-Based High Throughput Molecular Markers Targeting a Genomic Region Conferring Narrow Root Cone Angle in Aerobic Rice Production Systems
Source: Plants (Basel). 2021 Oct 3;10(10):2099. doi: 10.3390/plants10102099 (PMC8537842; doi:10.3390/plants10102099)

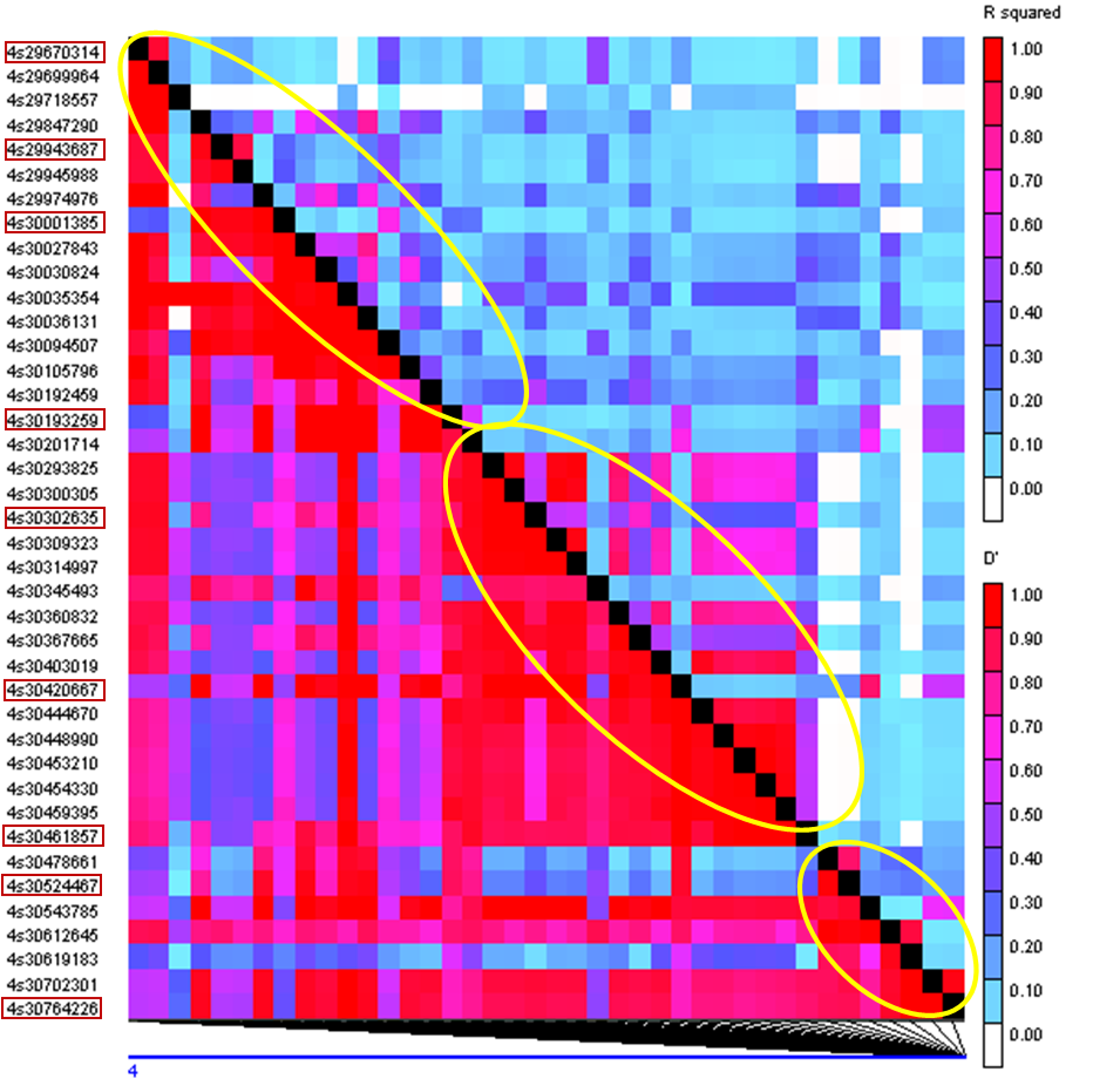

Supplement: Supplementary file 1 [file plants-10-02099-s001.zip › Fig. S1.png]

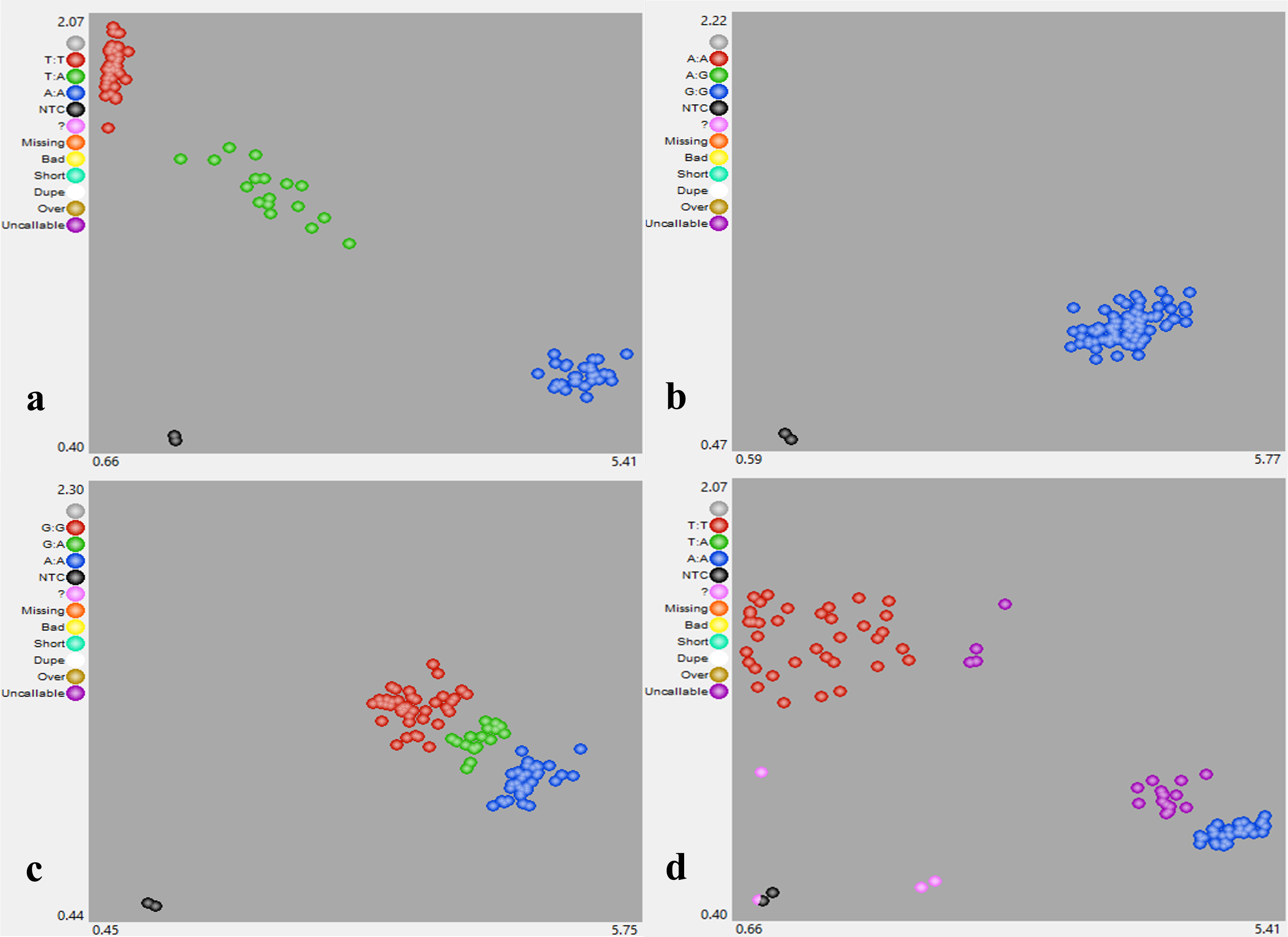

Supplement: Supplementary file 1 [file plants-10-02099-s001.zip › Fig. S2.png]
